# Defense-associated reverse transcriptases define an emerging class of noncanonical DNA-writing systems

**DOI:** 10.1016/j.synbio.2026.05.001

**Published:** 2026-05-20

**Authors:** Yaojun Tong

**Affiliations:** State Key Laboratory of Microbial Metabolism, School of Life Sciences and Biotechnology, Shanghai Jiao Tong University, Shanghai, 200240, China

**Keywords:** Defense-associated reverse transcriptase (DRT), Antiphage immunity, DNA synthesis, Noncanonical polymerization, Synthetic biology, Nucleic acid engineering

## Abstract

Defense-associated reverse transcriptases (DRTs) are emerging as a mechanistically diverse class of bacterial antiviral enzymes. Rather than acting simply as RNA-to-DNA copying proteins, they generate a wide range of unconventional DNA products, including de novo gene-assembling cDNAs, random single-stranded DNA, DNA homopolymers, poly(A)-rich cDNAs and protein-templated dinucleotide-repeat DNA. These findings suggest that DRTs are better viewed as DNA-construction modules than as unusual versions of canonical reverse transcriptases. In particular, DRT3 shows how RNA-templated and protein-templated synthesis can be combined within a single defence pathway to generate complementary repeat strands and repeat double-stranded DNA. We argue that this emerging chemistry expands the landscape of prokaryotic reverse transcription and may offer new reaction principles for synthetic biology and nucleic acid engineering.

## 1 Reverse transcription is no longer the right default description

Reverse transcriptases are usually introduced as enzymes that copy RNA into DNA. That description remains useful, but it is becoming inadequate for a growing set of bacterial antiviral systems. In just a few years, DRTs have expanded from a family-level curiosity into a mechanistically rich field in which distinct subfamilies generate different DNA outputs. DRT2 systems use non-coding RNAs to assemble de novo genes during phage infection [1,2]. DRT9 synthesizes long DNA homopolymers through an RNA-templated, protein-primed mechanism [3]. DRT3 now goes further by producing alternating dinucleotide-repeat DNA through a protein-templated process [4]. These studies suggest that DRTs are not simply unusual immune enzymes, but a broader repertoire of noncanonical DNA-writing systems.

This shift becomes clearer when the recent findings are placed in the longer context of prokaryotic antiviral RT biology. Gao and colleagues first systematically defined DRT1-9 as reverse-transcriptase-linked antiphage modules [5], and subsequent work on the UG/Abi lineage highlighted the remarkable diversity of RT-associated defense systems [6]. What was not yet clear was how different DRT families used DNA synthesis to mediate defense. That gap is now being filled by a wave of mechanistic studies.

## 2 One family, multiple DNA outputs

What now stands out is not just that DRT enzymes behave unusually, but that different DRT families generate different classes of DNA product through different reaction logics (Table 1). DRT2 provided the first clear example: two independent studies showed that a structured ncRNA drives reverse transcription to assemble a de novo toxic coding output during phage infection [1,2]. In this setting, reverse transcription is not merely copying. It is being used as a construction strategy.

**Table 1:** Current view of DRT1-DRT9 systems.

DRT type	Organizational mode	Core components	Experimentally supported activity	Current status	Refs.
DRT1	Family-level only	RT-associated antiphage module	Antiphage activity reported in original DRT survey	Dedicated mechanism not yet established	[5,6]
DRT2	ncRNA-containing	RT + structured ncRNA	Repetitive cDNA products assembling a de novo toxic coding output	Well established	[1,2]
DRT3	ncRNA-containing	Drt3a + Drt3b + ncRNA; D3-symmetric 6:6:6 complex	Alternating poly(GT/AC) dsDNA; Drt3a ncRNA-templated, Drt3b protein-templated	Well established structurally and biochemically	[4]
DRT4	Single-gene	Single RT protein	Template-independent random ssDNA; ORF55 protects 3′ end and promotes toxic accumulation	Well established	[7]
DRT5	Family-level only	RT-associated antiphage module	Antiphage activity reported at family level	Dedicated mechanism not yet established	[5,6]
DRT6	Single-gene, DRT4-related	Structural homolog of DRT4	ORF55 can also activate DRT6	Activation link supported; product chemistry less resolved than DRT4	[7]
DRT7	Multidomain RT fusion	RT-primase-polymerase (PP) fusion protein	Preprint evidence for protein-primed, protein-templated synthesis of palindromic poly(A)/poly(T)-rich duplex-like DNA	Mechanistic preprint available; peer-reviewed validation pending	[5,6,8]
DRT8	Family-level only	DRT subgroup defined in original survey	Antiphage activity reported at family level	Dedicated mechanism not yet established	[5,6]
DRT9	ncRNA-containing	RT + ncRNA; higher-order RT-ncRNA complex	RNA-templated, protein-primed poly-dA synthesis linked to abortive infection	Well established	[3]

A broader view suggests that DRT systems already divide into at least two organizational modes. One consists of ncRNA-containing systems, typified by DRT2, DRT3 and DRT9, in which the ncRNA functions as a structural and catalytic determinant of the active complex [[1], [2], [3], [4]]. The other consists of single-gene systems, typified by DRT4 and the related DRT6, in which the RT itself appears sufficient for synthesis, while phage-triggered stabilization of the DNA product converts synthesis into toxicity [7]. This division is not perfect, but it helps explain why DRT outputs are so diverse.

The products now span de novo gene-assembling cDNAs, random ssDNA, poly-dA homopolymers, poly(A)-rich cDNAs and patterned dinucleotide repeats [[1], [2], [3], [4],^7^,^9^]. Recent preprint work on DRT7 suggests that this repertoire may be broader still, describing an RT-primase-polymerase fusion that generates protein-primed, protein-templated, palindromic poly(A)/poly(T)-rich duplex-like DNA [8]. This diversity is difficult to reconcile with a view of DRTs as merely unusual copying enzymes. Instead, it points toward a family of DNA-construction systems repeatedly diversified by host-phage conflict.

## 3 How DNA synthesis becomes defensive

A central question is why making DNA can be defensive. DRT9 offered one strong answer. It produces poly-dA through a structured RT-ncRNA complex in which the ncRNA acts as both scaffold and template, while conserved tyrosines in the RT probably prime DNA synthesis [3]. Viral infection triggers poly-dA accumulation, which is linked to abortive infection and population-level immunity [3]. The same work connected this DNA product to interactions with phage and host DNA-binding factors, suggesting that DRT-derived nucleic acids may perturb replication-linked processes [3].

DRT4 reaches a related outcome through a different route. This single-gene system synthesizes random ssDNA in a template-independent manner [7]. During infection, the phage DNA-binding protein ORF55 protects the 3′ end of the DRT4-generated ssDNA from exonuclease degradation, allowing toxic ssDNA accumulation; ORF55 also activates DRT6, suggesting a shared trigger logic [7]. These findings support a broader model in which DRT-derived nucleic acids can act as toxic products or molecular sponges that sequester factors required for phage replication. Whether a similar logic applies to DRT3 remains unknown, but the idea that DRTs defend by producing DNA species that rewire the intracellular environment is already compelling.

## 4 DRT3 adds sequence control

**Fig. 1:**
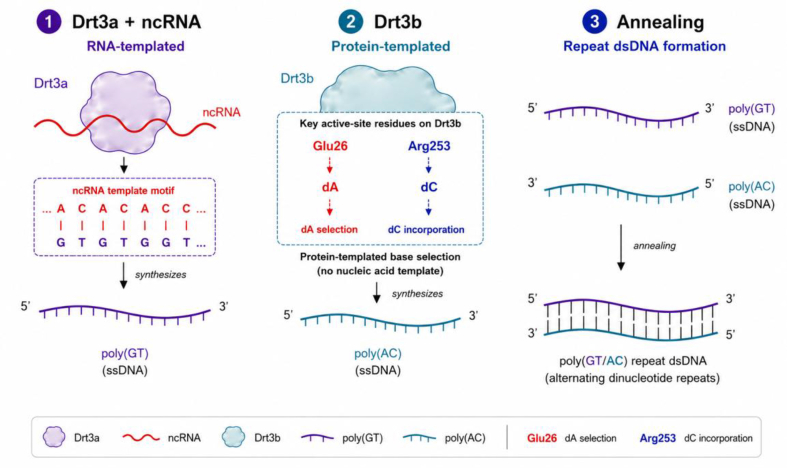
Core model of DRT3-mediated repeat DNA synthesis.

DRT3 is especially important because it adds a new level of sequence control. The recent Science study shows that DRT3 comprises two RT subunits, Drt3a and Drt3b, together with an ncRNA [4]. Drt3a uses a conserved ACACAC-like sequence within the ncRNA to produce poly(GT), whereas Drt3b synthesizes the complementary poly(AC) strand without a conventional nucleic acid template, yielding a final poly(GT/AC) double-stranded repeat product [4]. The two strands are therefore not generated by a single copying reaction. They are built by two distinct RT activities and then brought together into a paired product (Fig. 1).

The exceptional feature is that Drt3b does not polymerize randomly. Conserved active-site residues appear to enforce precise base alternation, allowing a protein-templated form of sequence-specific DNA synthesis [4]. The D3-symmetric 6:6:6 complex of Drt3a, Drt3b and ncRNA further indicates that DRT3 is not a loose assembly of catalytic parts but a highly organized molecular machine [4]. Its output is shaped not only by local chemistry, but also by the architecture of the whole ribonucleoprotein complex. This makes DRT3 an entry point into a wider design space in which sequence control can arise from active-site geometry and higher-order organization, not only from nucleic acid templates.

## 5 Why this matters for synthetic biology and biotechnology

Most familiar DNA-writing frameworks rely on template-dependent polymerases, template-independent transferases, recombination enzymes or CRISPR-linked integration systems. DRTs point to another space. Their outputs are shaped by nucleic acid templating, ncRNA architecture, oligomeric organization, protein priming, product stabilization and, in DRT3, direct protein-templated patterning [3,4]. None of these systems is yet a ready-made tool, but early enabling technologies rarely begin as tools. What matters first is whether they reveal new reaction principles. By that standard, DRTs already do.

The next phase should be comparative and synthetic. Which output features are programmable? How much is determined by RNA sequence, protein geometry or higher-order assembly? Can repeat identity, product length or trigger logic be redirected? DRT9 already suggests that some output features can be reprogrammed through template mutation [3]. If DRT3 proves similarly tractable, DRTs may move from fascinating antiphage biology toward a new enzymology for synthetic biology.

It would be tempting to frame these studies as a rewriting of the central dogma. That would be too strong. They do not demonstrate a direct protein-sequence-to-DNA-sequence information flow. But they do show that proteins can help determine how new DNA products are made and what repeat architecture emerges [3,4]. That is the more useful point. DRT3 may be less a final answer than a new entry point, suggesting that many additional nucleic acid synthesis modes remain hidden in nature. Recent preprint evidence from DRT7 further suggests that protein-templated DNA synthesis may extend beyond a single DRT family and may also emerge in multidomain RT systems that couple reverse transcription to additional catalytic activities [8]. For biotechnology, the importance of the field lies not only in bacterial immunity, but also in the possibility of discovering new reaction principles for programmable nucleic acid construction.

Drt3a synthesizes a poly(GT) strand from a conserved ncRNA template motif, whereas Drt3b generates the complementary poly(AC) strand through a protein-templated mechanism without a conventional nucleic acid template. Glu26 and Arg253 contribute to base selection in Drt3b. The two products anneal to form poly(GT/AC) repeat double-stranded DNA.

## Declaration of competing interest

The author declares that they have no known competing financial interests or personal relationships that could have appeared to influence the work reported in this paper. The author is an Associate Editor for Synthetic and Systems Biotechnology and was not involved in the editorial review or the decision to publish this article.
